# Design and performance of a bovine 200 k SNP chip developed for endangered German Black Pied cattle (DSN)

**DOI:** 10.1186/s12864-021-08237-2

**Published:** 2021-12-18

**Authors:** Guilherme B. Neumann, Paula Korkuć, Danny Arends, Manuel J. Wolf, Katharina May, Monika Reißmann, Salma Elzaki, Sven König, Gudrun A. Brockmann

**Affiliations:** 1 Animal Breeding Biology and Molecular Genetics, Albrecht Daniel Thaer-Institute for Agricultural and Horticultural Sciences, Humboldt Universität zu Berlin, Berlin, Germany; 2 Institute of Animal Breeding and Genetics, Justus-Liebig-Universität, Gießen, Germany; 3 Department of Genetics and Animal Breeding, Faculty of Animal Production, University of Khartoum, Khartoum North, Sudan

**Keywords:** Custom SNP array, Holstein cattle, Axiom MyDesign, Conservation, Breed-specific, Genetic reserve

## Abstract

**Background:**

German Black Pied cattle (DSN) are an endangered dual-purpose breed which was largely replaced by Holstein cattle due to their lower milk yield. DSN cattle are kept as a genetic reserve with a current herd size of around 2500 animals. The ability to track sequence variants specific to DSN could help to support the conservation of DSN’s genetic diversity and to provide avenues for genetic improvement.

**Results:**

Whole-genome sequencing data of 304 DSN cattle were used to design a customized DSN200k SNP chip harboring 182,154 variants (173,569 SNPs and 8585 indels) based on ten selection categories. We included variants of interest to DSN such as DSN unique variants and variants from previous association studies in DSN, but also variants of general interest such as variants with predicted consequences of high, moderate, or low impact on the transcripts and SNPs from the Illumina BovineSNP50 BeadChip. Further, the selection of variants based on haplotype blocks ensured that the whole-genome was uniformly covered with an average variant distance of 14.4 kb on autosomes. Using 300 DSN and 162 animals from other cattle breeds including Holstein, endangered local cattle populations, and also a *Bos indicus* breed, performance of the SNP chip was evaluated. Altogether, 171,978 (94.31%) of the variants were successfully called in at least one of the analyzed breeds. In DSN, the number of successfully called variants was 166,563 (91.44%) while 156,684 (86.02%) were segregating at a minor allele frequency > 1%. The concordance rate between technical replicates was 99.83 ± 0.19%.

**Conclusion:**

The DSN200k SNP chip was proved useful for DSN and other *Bos taurus* as well as one *Bos indicus* breed. It is suitable for genetic diversity management and marker-assisted selection of DSN animals. Moreover, variants that were segregating in other breeds can be used for the design of breed-specific customized SNP chips. This will be of great value in the application of conservation programs for endangered local populations in the future.

**Supplementary Information:**

The online version contains supplementary material available at 10.1186/s12864-021-08237-2.

## Background

German Black Pied cattle (DSN, Deutsches Schwarzbuntes Niederungsrind) are a dual-purpose breed that has been selected for milk and beef production. The breed that originally comes from the Dutch and German North Sea region is considered an ancestor population of the modern Holstein dairy cattle breed. While the modern Holstein breed became the most commonly used breed for milk production worldwide, DSN cattle were kept as a genetic resource population without strong selection and maintained its dual-purpose character. However, since DSN cows produce about 2500 kg less milk per lactation compared to Holstein cows, a high replacement rate of DSN by Holstein cattle decreased the DSN herdbook to about 2500 animals over the last 30 years [1] leading to its classification as an endangered breed [2]. Since 1989, DSN has been kept as a genetic reserve through financial support from the German government. The goal is to maintain DSN as a pure breed with improved milk production in an affordable small population. To conserve its genetic diversity and maintain the DSN-typical characteristics in the breeding program, it is crucial to study its genetics at the populational level.

In the past, the commercially available Illumina BovineSNP50 BeadChip® (Illumina Inc., San Diego, CA) (Illumina BovineSNP50) was used to genotype DSN cattle for genome-wide association studies (GWAS) [3–7]. This chip contains about 36.9 k SNPs informative in DSN after filtering for a SNP call rate > 95% and a minor allele frequency (MAF) > 5% [7]. The loss of up to 20 k SNPs due to filtering has also been reported in other studies on small-breeds [8–11]. The loss can be even more extreme, if a commercial SNP chip is used for distantly related species (for example, *Capra ibex* when analyzed using the commercially available Illumina GoatSNP50 chip [12]). In comparison, for the Illumina BovineSNP50 around 10 k SNPs are unsuitable in big commercial breeds [13–17]. This observation is unsurprising since the BovineSNP50 BeadChip from Illumina as well as the Axiom Bovine Genotyping Array from Thermo Fisher Scientific (Massachusetts, USA) were designed for commercial breeds such as Angus, Simmental, Jersey, Holstein, and Hereford [18, 19] and not all of the SNPs were segregating in a single commercial breed. Inherently, informative, and breed-specific variants segregating in various small and/or local breeds, such as DSN, were not considered. This leads to a marker ascertainment bias [20, 21] that does not account for haplotypes segregating in smaller breeds (e.g. out of 186,204 haplotype blocks detected in DSN using whole-genome sequencing data, only 28,712 were covered by polymorphic SNPs of the Illumina BovineSNP50), and informative markers might not be equally distributed across the genome, which could cause wrong/weak conclusions in genetic relationship studies. Moreover, the accuracy of estimated breeding values of a specific breed is reduced when they are based on another reference population [22, 23]. Therefore, SNP chips designed for big commercial breeds are not optimal for diversity studies and genetic evaluations and cannot be used to maintain breed-specific genetic features. For that reason and with the focus on managing and maintaining the endangered DSN population as a valuable genetic resource, we developed a customized SNP chip that considers genetic variants unique to DSN in addition to informative SNPs from the Illumina BovineSNP50 and genetic information from additional breeds.

Genome-wide SNP chips are widely used for many livestock species and are a cost-effective alternative to whole-genome sequencing (WGS) and to Genotyping by Sequencing. The output of the latter, even though it may seem like a cheaper option, presents lower genotype quality and a lot of missing data. Moreover, it also fails to target many haplotype blocks since only a limited amount of primers are used to define target regions [24]. For that reason, in addition to commercially available SNP chips, custom SNP chips benefit local breeds, providing information regarding important haplotype blocks completely absent in commercial chips. Specific variants on customized SNP chips are particularly interesting for the maintenance of breed-specific genomic variants and properties of different small populations with a specific genealogy.

The two most widely used genotyping technologies are the Illumina Bead chip (Illumina Inc., San Diego, California, USA) and the Affymetrix Axiom genotyping array (Thermo Fisher Scientific, Waltham, Massachusetts, USA). In both cases, multiple oligonucleotides (probes) for each variant are attached to the surface of the chip. In the case of Affymetrix SNP chips, the SNP is represented by two 71-mer probes (probeset) each targeting one possible sequence variant, whereof the target allele is surrounded by 35 bp up- and downstream. An exception are SNPs carrying A/T or G/C variants, which cannot be detected by the two-fluorescence dye labeling system for G and A nucleotides and, therefore, require an additional probeset. In the case of indels, the same technique is applied, where one probe contains the deletion while the other does not. The sample DNA then binds to one of the probes, resulting in the specific fluorescent signal [25, 26]. In the case of Illumina SNP chips, each variant is represented by a single probe attached to a bead; the 50-mer probe ends one base upstream the target variant. After DNA binding, the detection of the allele then occurs through the extension of the probe by a fluorescent-marked nucleotide. Similar to Affymetrix, Illumina’s technology is also a two-color system and therefore, A/T and G/C variants are detected by two probes [27]. Further differences, which affect the decision for one technology or the other, are concerning the chip format. In both cases, the chip varies in sample capacity, directly affecting final prices. For the small DSN population, Affymetrix was the most flexible platform and had the best cost-benefit ratio with regard to the number of SNPs on the chip and the amount of samples to be genotyped.

Here, we showcase the newly developed bovine DSN200k SNP chip representing 182,154 sequence variants comprising 173,569 SNPs and 8585 indels. The chip was developed using the Axiom array technology (ThermoFisher Scientific, Massachusetts, USA). We verified the performance of the DSN200k SNP chip on DSN, Holstein, and other endangered local cattle populations, including Rotes Höhenvieh, Angler, Rotbunt DN, Hinterwälder, Gelbvieh, Original Braunvieh, Pinzgauer, and even for Butana cattle as a *Bos indicus* breed.

## Results

### DSN sequence variants from whole-genome sequencing

For the design of the DSN200k SNP chip, a representative dataset of DSN sequence variants was needed. Therefore, 304 DSN animals were systematically selected for whole-genome sequencing (WGS) and further variant discovery. After alignment of data against the *Bos taurus* genome assembly ARS_UCD1.2_Btau5.0.1Y [28, 29], 20,586,171 high-quality variants comprising 18,556,008 SNPs and 2,030,163 indels were identified (Table S1). Concordance rate of 99.60 ± 0.41 was found comparing results from our pipeline with calls for 57 DSN included in the 1000 Bull Genomes project (Run 8) which were overlapping between the two datasets.

Sequence variants predicted with severe potential effects were of interest to our design. For that reason, potential effects of the 20,586,171 variants on transcripts were predicted using the Ensembl Variant Effect Predictor (VEP) [30] (Table S2). Most variants (97.17%) were labeled as “modifier”, which are usually non-coding variants or variants affecting non-coding genes. “Low impact” variants (2.19%) were mostly counting for synonymous variants, while “moderate impact” variants (0.60%) were mainly missense variants. The variants predicted with the most severe consequences were labeled “high impact” (0.04%). Those categories of impact were used to partially guide the selection of variants for the DSN200k SNP chip.

### Sequence variants on the DSN200k SNP chip

The DSN200k SNP chip is a custom Axiom® myDesign TG Array technology (Thermo Fisher Scientific, Massachusetts, USA) with a capacity of about 200 k probesets. The final DSN200k SNP chip harbors 182,154 variants comprising 173,569 SNPs and 8585 indels selected based on 10 categories (see Methods). These variants cover 103,801 out of 186,204 estimated haplotype blocks in DSN. The number of probesets that was necessary to present the 182,154 variants on the chip sums up to 203,511. A visualization of the number of selected variants per chromosome for each selection category is provided in the Supplementary Information (Fig. S1). The complete list of variants is provided in Table S﻿3.

**Fig. 1:**
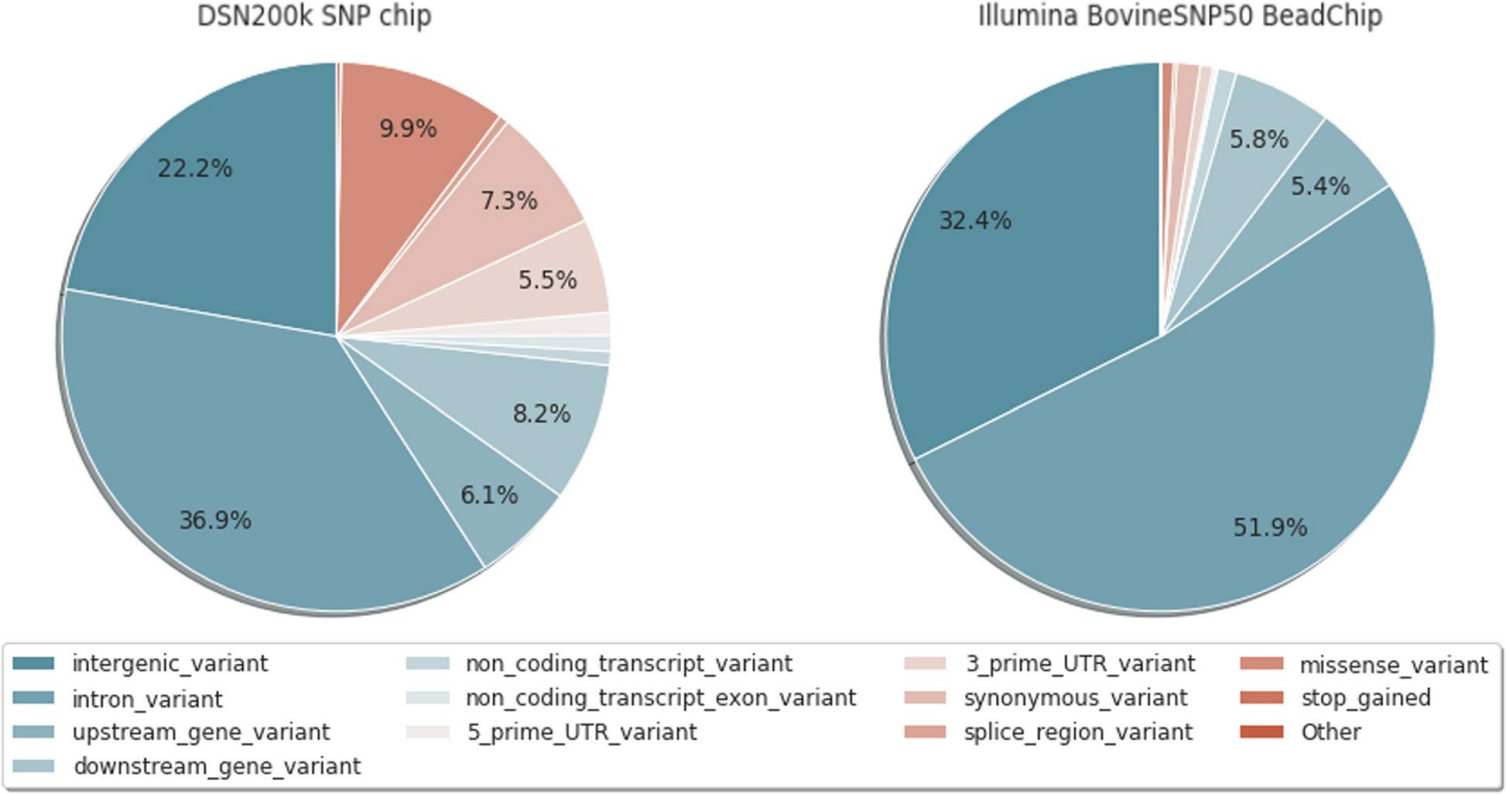
Comparison of variant effects between the DSN200k SNP chip and the Illumina BovineSNP50 BeadChip using the Ensembl Variant Effect Predictor (VEP). The color indicates the impact of each consequence from the least severe (blue) to the most severe (red)

Altogether, SNPs of the DSN200k SNP chip had an overlap of 49,569 SNPs and 35,025 SNPs with the Illumina BovineHD BeadChip (Illumina Inc., CA, USA) and the Illumina BovineSNP50 BeadChip v.3, respectively. Those 35,025 SNPs from the Illumina BovineSNP50 are not only derived from category 1 (Illumina BovineSNP50 DSN informative) but also from other selection categories. A comparison of functional annotation between the DSN200k SNP chip and Illumina BovineSNP50 shows the differences in their design goals. This specific comparison to the Illumina BovineSNP50 and not to other chip formats or densities is important due to the wide use of the Illumina BovineSNP50 in bovine studies, including previous DSN studies. 9.9% of the DSN200k SNP chip is composed of missense variants and 7.3% of synonymous variants but only 0.7 and 0.4% of the Illumina BovineSNP50, respectively (Fig. 1). This reflects the nature of our design, which prioritized higher impact predicted consequences over modifier variants.

It has to be noted that the number of unique variants – meaning variants that were exclusively selected in a certain category given the established order – is different from the total number of variants per category (Table 1) due to overlaps between categories of selection. For example, the total number of variants in category 2 (Associated with traits of interest (GWAS)) was 2071 variants, but 135 SNPs of these variants were already selected based on category 1 (Illumina BovineSNP50) so that the unique number of variants based on the selection category 2 was 1936 variants.

**Table 1:** Number of unique, total, successfully called (high-quality genotype calls), and in the population segregating variants (SNPs and indels) on the DSN200k SNP chip per category of selection

Categories of selection	# Unique variants^a^	#Total variants^b^	#Successfully called unique variants in DSN (***n*** = 300)	#Successfully called unique variants across breeds (***n*** = 462)	#Segregating unique variants (MAF > 0.01) in DSN
1) Illumina BovineSNP50 DSN informative	34,039	34,039	32,996 (96.94%)	33,650 (98.96%)	32,973 (96.87%)
2) Associated with traits of interest (GWAS)	1936	2071	1790 (92.46%)	1869 (96.54%)	1772 (91.53%)
3) High, moderate, or low impact	49,177	50,611	44,685 (90.87%)	46,419 (94.39%)	43,032 (87.50%)
4) DSN unique	37,388	38,198	32,579 (87.14%)	33,348 (89.19%)	25,903 (69.28%)
5) High difference in alternative allele frequency between DSN and Holstein	49	55	44 (89.80%)	46 (93.88%)	44 (89.80%)
6) Y chromosome	321	321	276 (85.98%)	279 (86.92%)	3 (0.93%)
7) Mitochondria	278	278	258 (92.81%)	258 (92.81%)	22 (7.91%)
8) Parentage panels	64	554	63 (98.44%)	63 (98.44%)	62 (96.88%)
9) Haplotype blocks	58,886	103,801	53,863 (91.47%)	55,855 (94.85%)	52,864 (89.77%)
10) Filing gaps > 250 kb	16	16	9 (56.25%)	11 (68.75%)	9 (56.25%)
**Total**	**182,154**		**166,563 (91.44%)**	**171,798 (94.31%)**	**156,684 (86.02%)**

^a^Unique variant refers to variants selected per category of selection without overlaps, given the selection order

^b^The total number includes all variants per category, independently of selection order

**Fig. 2:**
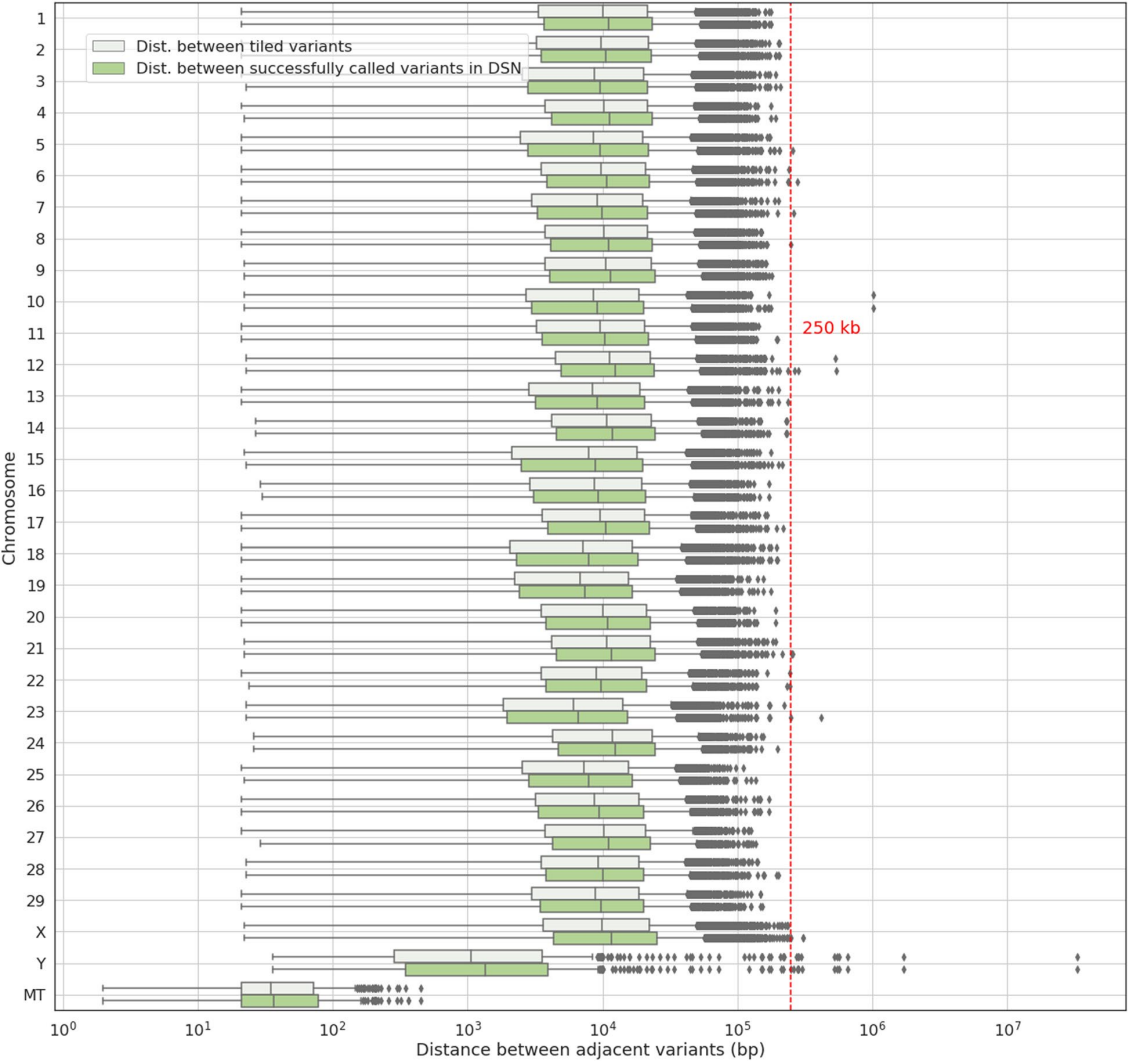
Distribution of the physical distances between adjacent variants tiled on the DSN200k SNP chip per chromosome. Boxplots are shown for all variants on the DSN200k SNP chip (light green) and successfully called variants in all 300 genotyped DSN animals (green). The red line indicates the maximum distance of 250 kb aimed for during chip design. Two gaps longer than 250 kb exist on chromosomes 10 and 12, and ten gaps on the Y chromosome that could not be filled with variants. New gaps longer than 250 kb but shorter than 500 kb appeared due to failing variants after genotyping

On average, the DSN200k SNP chip contains one variant every 14.4 kb on the autosomes. The average distances between variants on the X, Y chromosome and mitochondria are 16.7 kb, 127.7 kb and 57.9 bp, respectively. The maximum distance between neighboring variants on the autosomes and X chromosome does not exceed 250 kb (Fig. 2), except for one gap on *Bos taurus* autosome (BTA) 10 (23,775,405 - 24,782,658) and one on BTA 12 (71,953,410 - 72,484,697) where no variants were detected in DSN. In contrast, big gaps are located on the Y chromosome (2,610,120 - 4,303,972; 9,259,296 - 42,160,160) where no variants were available or recommended during the chip design.

### Performance of the DSN200k SNP chip for DSN and other breeds

The performance of the DSN200k SNP chip was first evaluated with 300 DSN animals. After quality filtering and genotype correction, out of the 182,154 variants (173,569 SNPs and 8585 indels) on the chip, 166,563 (91.44%) variants consisting of 159,622 (91.96%) SNPs and 6941 (80.85%) indels were successfully called (Table 1). Among those variants, 156,684 were segregating in DSN at a MAF > 0.01. If we considered all breeds, DSN and the other breeds genotyped (*n* = 462), 10,356 (5.69%) variants comprising 9204 (5.30%) SNPs and 1152 (3.42%) indels failed. The percentage of variants that did not call properly ranged between 31.25% (filling gaps > 250 k) and 1.14% (Illumina BovineSNP50 DSN informative) among the 10 categories of SNPs for the chip (Table S4).

**Fig. 3:**
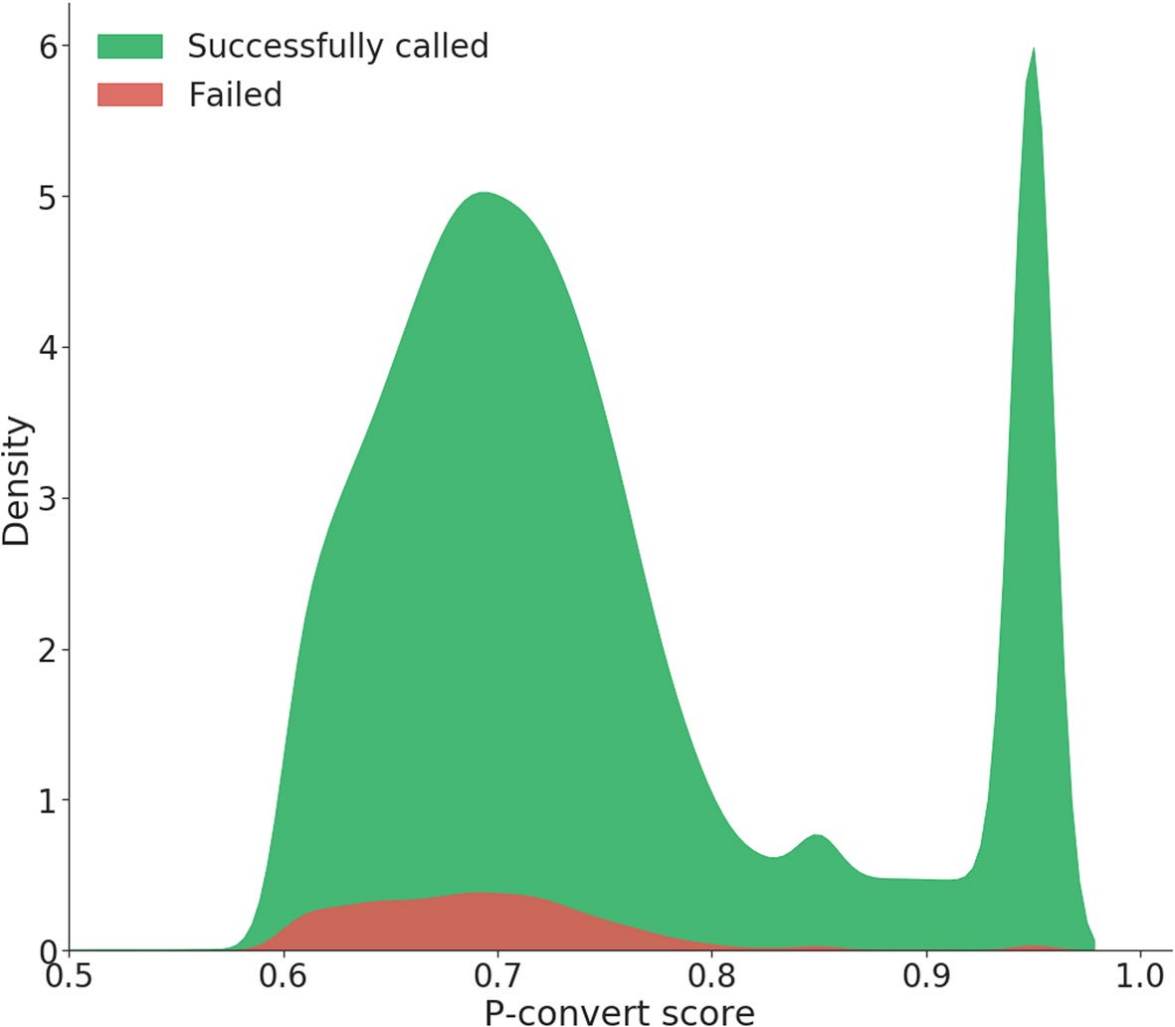
Distribution of p-convert scores derived from in silico analysis for successfully called and failed variants tiled on the DSN200k SNP chip used for 462 animals from 10 different taurine breeds

In total, 2071 variants are on the DSN200k SNP chip that were associated with traits of interest in DSN (1936 selected in category 2). Since those variants were represented on the chip with multiple probesets (unless they were derived from imputed WGS data), variants from this category called successfully (92.46%) above the average of all variants (91.44%). The variants from GWAS that failed were mainly derived from studies with imputed WGS data (66 variants compared to only 1 non-imputed SNP). DSN unique variants had a slightly lower success rate (87.14%). For the Y chromosome, 276 out of 321 (85.98%) SNPs were called, but only 3 of them were segregating in DSN and 15 across all investigated breeds (Table S5). With respect to mitochondria, it was our goal to add more SNPs than only the 14 detected and recommended in DSN, because variation in the mitochondrial genome is informative for diversity studies, in particular for the maternal side. Therefore, we used all mitochondrial SNPs available from Thermo Fisher Scientific’s database which increased the risk of having non-working and non-segregating SNPs in this category. The drop-out of different types of variants finally caused eight more gaps longer than 250 kb (but shorter than 1 Mb – Fig. 2) in the DSN genome which are located on BTA 5 (66,816,466 – 67,070,552), BTA 6 (32,711,954 – 32,991,881), BTA 7 (49,420,054 – 49,678,444), BTA 12 (38,498,722 – 38,763,631; 72,592,348 – 72,875,145), BTA 21 (1,276,150 – 1,529,357; 3,041,574 – 3,296,627), and BTA 23 (25,920,341 – 26,334,918). Most variants that failed calling had a p-convert score (an in silico value for the potential technical performance of a variant on the chip) of around 0.6 and 0.7, although the minimum threshold of 0.6 was suggested by the manufacturer (Fig. 3). The mean p-convert from failing variants varies, however, between the categories of selection (Table S4) reflecting the prioritization of higher scores in the haplotype block selection category.

In a second quality test, we compared the accuracy of DSN200k SNP chip genotype data of 15 DSN animals with their genotypes obtained from WGS. The concordance rate was 99.09 ± 0.28%. Furthermore, the repeatability with 16 cattle that were genotyped multiple times with the DSN200k SNP chip in different batches, showed a concordance rate of 99.83 ± 0.19%, technically validating the chip.

In addition, we tested the performance of the DSN200k SNP chip with 20 Holstein cattle, 137 cattle from other German endangered breeds (30 Rotes Höhenvieh, 30 Angler, 30 Rotbunt DN, 2 Hinterwälder, 12 Original Braunvieh, 23 Pinzgauer, 10 Gelbvieh), and 5 Butana cattle as outliers (Table S5). The number of successfully called variants in at least one breed was 171,798 (94.31%). Surprisingly, the highest number of successfully called variants was evident in Pinzgauer (169,125; 92.85%) followed by Holstein (166,967; 91.66%) which both had more called variants than DSN. The lowest numbers of successfully called variants were found in Gelbvieh (154,900; 85.04%), Original Braunvieh (158,151; 86.82%), Butana (158,160; 86.83%), and Rotes Höhenvieh (158,813; 87.19%).

Unsurprisingly, the number of segregating and, therefore, informative variants was highest in DSN (156,684; 86.02% - Table 1). The frequency of informative variants in most other breeds ranged from 48.34 to 66.11% in Butana and Pinzgauer, respectively (Table S5). An exception was Hinterwälder with 21.90% informative variants. The low frequency of informative variants in Hinterwälder results from the low number of two genotyped animals, only, since the number of segregating variants is directly related to the number of genotyped animals. Due to the high number of DSN unique variants on the chip (37,388) which are key to the management of diversity and the study of important traits in DSN, a high number of non-segregating variants in other breeds than DSN was expected.

The number of variants that was successfully called in five *Bos indicus* Butana cattle was 158,160 (86.81%) comprising 151,583 (87.33%) SNPs and 6577 (76.61%) indels. Among them, 88,056 (48.34%) variants (85,231 SNPs (49.10%) and 2825 (32.91%) indels) were segregating. The comparison of genotypes obtained from the SNP chip and those from WGS data for these five Butana cattle provided a concordance of 98.30 ± 0.29%.

## Discussion

The current paper introduces a customized SNP chip designed for the dual-purpose cattle breed DSN and evaluates the developed chip in DSN and other endangered cattle breeds in Germany. The selection criteria implemented in our work are not commonly seen in other SNP chip designs. Generally, the in silico conversion, physical or genetic distance between markers, and allele frequencies are the main selection motives [31–34]. Nevertheless, a similar approach prioritizing high impact and/or rare variants, and candidate variants to important traits as we implemented was also used for the catfish 250 k array [35], GeneSeek Genomic Profiler™ F250 chip [36], and 50 k Axiom bald eagle SNP chip [37]. In our case, we were able to select informative variants in DSN and keep a uniform distribution across the genome in DSN including variants targeting all haplotype blocks longer than 5 kb, whereof most were even longer than 1 kb.

The only exception for long genomic stretches without variants was found on the Y chromosome. The intention to include SNPs on the Y chromosome would not only create the possibility of gender computation but would also be an advantage over other SNP chips, where the Y chromosome is not commonly present. However, the lack of knowledge regarding the bovine Y chromosome poses a challenge when predicting mutations and/or dealing with pseudo-autosomal regions (PAR). Only recently, the PAR region on the X chromosome has been defined [38], but little is known about the bovine Y chromosome PAR region. The variants very likely located within the PAR showed low coverage, and the few variants that passed the coverage threshold (470 reads, since 47 bulls were sequenced) failed the in silico conversion. Therefore, there is a lack of variants between 9 Mb and 42 Mb on the Y chromosome. Extra SNPs on the X and Y chromosomes, however, are still internally present on the chip, and are used by Thermo Fisher Scientific to verify gender.

The total success rate of 94.31% across all breeds, and the segregating rate of 86.02% in DSN, was high in comparison to other described works with the Affymetrix technology, varying, for example, from a calling rate of 46.16% in the Atlantic salmon [39], 74.81% in water buffalo [40], to 90.48% in the bald eagle [37]. The decision to use SNPs that were informative on the Illumina BovineSNP50 was straightforward not only because these SNPs were successfully called but also because these SNPs, which are common on both chips, allow the joint analysis of animals genotyped with the Illumina BovineSNP50 and the DSN200k SNP chip.

The analysis of potential reasons why variants failed calling despite careful selection points at deviations from ideal selection thresholds as the main source. For example, not all DSN informative SNPs from the Illumina BovineSNP50 were called on the new chip in genotyped DSN (96.94%). In more detail, among the 389 DSN informative SNPs from the Illumina BovineSNP50 that failed on our chip, 191 SNPs had another variant in the sequenced DSN cattle between 20 and 35 bp up- or downstream of the target SNP position. A distance of 20 bp was our exclusion criterion for choosing SNPs for the chip on this category (see Methods for more details). The reason is likely the difference in SNP chip technology between Illumina and Affymetrix, which have different locations of the target variant in the probe sequence: middle of the probe (36th bp) or last base of the probe (51st bp) on Affymetrix and Illumina, respectively. In addition, it is possible that other interfering variants that were not detected in those 304 animals may exist in the DSN population, and even more likely in the other studied breeds, affecting DNA hybridization. However, this difference alone does not explain the failing call rates fully.

The lower calling rate in the DSN unique category (87.14%), for instance, is in part the results of an insufficient readout of genotypes after chip hybridization (Table S4). Since most of the DSN unique variant alleles are rare, they form small unsharp genotype group clusters. This difficulty is expected to reduce when genotyping many more animals so that more alleles contribute to the allelic cluster. Even though rare variants are challenging, those play an important role in diversity analysis, mitigating ascertainment bias [21], and for that reason, were kept during the chip design. Besides that, rare variants are difficult to reconstruct through imputation, which make them valuable when called on SNP chips [41].

Moreover, some sequencing and alignment errors, mostly removed by the Variant Recalibration tool from GATK as recommended by the 1000 Bull Genomes Project, could still be present in the dataset (Fig. S2). Therefore, a stricter tranche, training and truth datasets specific for DSN, or a standard hard filtering may be implemented for filtering when selecting alternative variants in future updates of the DSN200k SNP chip.

The reasons for failing genotyping of variants on a SNP chip can depend on the selected technology. As discussed, interfering variants might cause improper hybridization [42]. Since probes on the Illumina platform consist of oligonucleotides upstream of the target variant, interference from downstream variants cannot occur. Further, the 50 bp long probe sequences make hybridization more stable in the presence of interfering variants, in contrast to 35 bp long probe sequences up- and downstream which are used by Affymetrix. In addition, Illumina uses shorter probe sequences, which in comparison to longer probes as used by Affymetrix are less likely to be unique in the studied genome. However, those differences are not much of a concern since variants fail to be called in both technologies for many different reasons [42]. As recently discussed for human genetics [43], the content of a genotyping chip is more important than the technology applied.

Nonetheless, we are confident that the 166,563 variants selected for the DSN200k SNP chip and working in DSN represent the majority of DSN’s diversity, accomplished through the idea of linkage between variants. 97,187 haplotype blocks (out of 103,801 tiled in the chip) were called in DSN animals, covering most of the high, moderate, and low predicted impact variants detected in sequenced DSN. Out of 131,341 variants detected in DSN sequenced animals predicted to have high or moderate impact, we find a total of 112,389 located inside a haplotype block which holds a selected variant on the chip. The same for the low impact variants, out of 455,486, 400,561 are covered by the chip due to another selected variant. There were not many failing variants in the category with high, moderate, or low predicted impact also happening in other categories of selection. For instance, out of 38,198 DSN unique variants (Table 1), only 35 failed with high or moderate impact and 78 with a low impact. Out of 34,039 Illumina BovineSNP50 DSN informative variants, 11 were failing with a high or moderate impact, and 45 with a low impact. Out of 2071 variants associated with traits of interest, only 1 which failed had a low impact. And out of 554 variants from parentage panels, 2 variants were failing with low impact. The total of 15,591 variants which failed in DSN are neither causing long gaps (Fig. 2) nor causing gaps in genetic distance defined by linkage disequilibrium. An average D-prime of 0.97 ± 0.07 and R^2^ of 0.73 ± 0.26, as calculated by PLINK v1.9, were observed between failing and working variants for DSN.

Moreover, the chip can be used for other local breeds. Even though the DSN unique variants are monomorphic in these breeds, the use of the whole DSN200k SNP chip or of single polymorphic variants from the chip is possible (see Table S3). For example, the validated variants can be used to generate another customized chip for other local breeds, and even for Butana. Nevertheless, additional WGS data of more animals would be needed to identify variants that cover the genome of other breeds. Besides, breeds that are closer related to DSN, such as Holstein, Angler, Rotbunt DN and Pinzgauer could benefit from the DSN200k SNP chip.

Some of the variants classified as DSN unique were found segregating in other breeds. That is due to the reason that the detected variants in DSN were only compared to certain cattle populations, which in turn did not cover all breeds used to validate the DSN200k SNP chip and which were also represented only by a limited number of individuals.

For the production of customized chips, we recommend applying rigorously highest quality requirements. These include for the used Axiom® myDesign TG Array technology: avoid variants in the close neighborhood of at least 35 bp of the target SNP position, prefer SNPs over indels, choose variants with the highest p-convert score possible. The 10,356 variants failing in all breeds, which mainly stem from the categories DSN unique (4040), haplotype block coverage (3031), and predicted impact (2758) should be replaced in a next version of the DSN200k SNP chip, selecting variants with higher p-convert values and higher minor allele frequencies. This is an advantage of working with customized chips. Based on this first validation, and on the list of in silico scored probes detected in DSN, next releases of the DSN200k SNP chip will be improved so that we expect better call rates. Variants presenting low allele frequencies, thus being less informative, might be replaced, eventually applying a scoring procedure based on MAF as implemented in the 90 k buffalo SNP chip [40]. Better focus on regulatory elements and alternative functional annotation could be applied. The same is conceivable for the application of the chip for other breeds, replacing DSN unique variants with variants segregating in target populations. This is especially interesting to breeds closely related to DSN, such as Rotbunt DN.

## Conclusions

With the DSN200k SNP chip, we developed a chip that can be applied in DSN for the management of the small population. The genotype information can be used for targeted mating to prevent inbreeding and maintain alleles that are unique to the breed and to increase the frequency of alleles that might be favored for the development of the breed as a niche product. In particular, the chip is useful for genotyping of potential breeding bulls to support the selection of most favorable candidates. Although the chip was primarily developed for DSN cattle, the variants on the chip are informative in a wide range of cattle breeds, including the *Bos indicus* Butana cattle. Thus, the chip can be used for genotyping of divers *Bos taurus* as well as *Bos indicus* breeds.

## Methods

### Whole-genome sequencing data

#### Selection of animals for sequencing

304 DSN animals were selected for WGS. The selection included all breeding sires for which any cell material was available (*n* = 47) as well as cows from different farms. A systematic selection of cows was applied, considering all subfamilies and extremes values for traits such as milk yield and milk composition, breeding values for reproduction, and health diagnoses (mastitis and endoparasite). The number of cows selected per sire was adjusted to the total number of offspring per sire. Traits, breeding values and pedigree data was provided by VIT (Vereinigte Informationssysteme Tierhaltung w.V.); health data was obtained from the DSN breeding organization (Rinderproduktion Berlin-Brandenburg GmbH) and from prior projects with focus on DSN.

#### Sequencing of DSN and variant discovery

Paired-end sequencing of the DSN cattle was performed on the Illumina NovaSeq platform 150 PE. The coverage was on average 19-fold, with 18.72-fold ±2.44 coverage after trimming and mapping, ranging from 7.75 to 26.25 per animal. Data pre-processing, sequence read mapping, variant discovery and recalibration were performed following the 1000 Bull Genomes Project guidelines (Hayes & Daetwyler, 2018) using the *Bos taurus* genome version ARS-UCD1.2_Btau5.0.1Y as reference [28, 29]. The recalibration was done for SNPs and indels separately using VariantRecalibrator and ApplyVQSR in GATK v.4.1.3.0 [44]. Training and truth set variants for *Bos taurus* were provided by the 1000 Bull Genomes Project (Run 8). The filtering in the software GATK was done based on tranches, in which each tranche represents the percentage of variants from the truth set retained, and consequently the number of predicted true-positive and false-negative variants which were also retained. We selected the 99% tranche as threshold in order to retain as many true-positive variants as possible while removing most false-negative variants (Fig. S2). For the validation of our variant calling pipeline for DSN cattle, we calculated a concordance rate with 57 out of the 304 sequenced DSN animals which had been submitted to the 1000 Bull Genomes project (Run 8).

#### Identification of DSN unique variants

In order to identify DSN unique variants, alternative allele frequencies (AAFs) were calculated for DSN and genetically closely related breeds (Holstein, Modern Danish Red, Original Braunvieh, Gelbvieh, Ayrshire Finnish, Normande, Norwegian Red, Swedish Red, and Jersey) available in the 1000 Bull Genomes Project (Run 8) using vcftools v0.1.16 [45]. Sequence variants found in DSN were defined as DSN unique if they are present in DSN (AAF_DSN_ > 0.01) but absent in genetically closely related breeds (AAF = 0).

#### Annotation of variants using VEP

Variants identified in DSN were annotated on the bovine genome ARS-UCD1.2 (Y chromosome absent) using VEP software [30]. The effect of a variant was determined based on known effects from the Ensembl database and predictions of functional consequences implemented inside VEP by the SIFT algorithm [46]. We defined the consequence types “coding sequence variant”, “mature miRNA variant”, “5’ UTR variant”, “3’ UTR variant”, and “non-coding transcript exon variant” as “low impact”, instead of modifier as stated by VEP.

#### Haplotype block construction

Haplotype blocks were calculated based on all sequenced DSN animals following the standard definition of the Human Genome Project [47] using PLINK v1.9 [48]. Haplotype blocks were defined within windows of 200 kb where all contained variants are in linkage with each other given a distribution of linkage values (D-prime) with a 90% bottom confidence interval ≥ 0.60 and top confidence interval ≥ 0.85.

### SNP chip design

#### Filtering of variants for technical suitability

The DSN200k SNP chip is based on the Axiom® myDesign TG Array technology (Thermo Fisher Scientific, Massachusetts, USA), which allows to use both SNPs and indels. All biallelic high-quality variants identified for DSN (20,586,171) were first filtered for their technical suitability by constructing unique oligonucleotides of a length of 71 bp for SNPs and longer oligonucleotides for indels [49], before variants would be selected for their biological information content.

To ensure high specificity of allele calls and to prevent interfering effects of variants in the close neighborhood of variants, all variants which have another variant within 35 bp up- or downstream were removed. For important selection categories (category 2, 3, and 4, see below under “Selection of informative variants”), we allowed no variant to be located within 20 bp (compared to 35 for less important categories) allowing an interfering variant located 20 bp up- or downstream of the target variant. After this filtering step, 8,903,849 out of the initial 20,586,171 variants were left.

Afterwards, variants with a read coverage below 3000 across all animals, corresponding to an average coverage of less than 10 reads per animal, were removed. This resulted in the removal of additional 253,804 variants, leaving 8,650,045 variants.

Variants with AAF in DSN (AAF_DSN_) below 0.05 were removed, except, if variants were labeled as “DSN unique”, or AAF in Holstein (the closest related breed to DSN), or Original Braunvieh or Gelbvieh was higher than 0.05, and AAF_DSN_ > 0.01. The consideration of other breeds than DSN was necessary to test potential future applications of the new chip for other endangered or dual-purpose breeds and to allow comparison of DSN to Holstein.

Finally, Thermo Fisher Scientific scored the variants for technical recommendation for the DSN200k SNP chip using an in silico scoring algorithm based on their empirical database. The resulting p-convert score which was calculated for each variant and each strand refers to a conversion rate of a variant based on interfering polymorphisms, repetitive regions, probe’s identity, and empirical conversion rate known from other chips. Variants with a p-convert score < 0.6 for both strands were removed in this filtering step, leaving 3,069,815 variants that passed this technical suitability check.

#### Selection of informative variants

Among technical suitable variants, we selected the most informative variants in terms of molecular function, uniqueness, association to important traits, and differentiation to other breeds, following 10 selection categories in the given order:

1. Illumina BovineSNP50 DSN informative: SNPs from the Illumina BovineSNP50 informative in DSN (SNP call rate ≥ 0.95 and MAF ≥0.05 in DSN based on data from previous studies [6, 7]);
2. Associated with traits of interest (GWAS): Variants significantly associated with diverse traits of interest in DSN resulted from Illumina BovineSNP50 and imputed data. Significant variants were available for milk traits [7], clinical mastitis resistance [6], endoparasite resistance [3], heat stress [50], breeding values for exterior and fertility (not yet published);
3. High, moderate, or low impact: Variants with high, moderate, or low impact effect predicted by VEP;
4. DSN unique: Variants that were detected unique in DSN;
5. High difference in alternative allele frequency between DSN and Holstein: Variants with a difference of the AAF higher than |0.70| between DSN and Holstein;
6. Y chromosome: All variants detected in DSN on the Y chromosome;
7. Mitochondria: All variants detected in DSN and 264 available from the Axiom’s database located on the mitochondria;
8. Parentage panels: All variants from the parentage panels suggested from the International Society for Animal Genetics and the International Committee for Animal Recording [51, 52]. These variants are highly diverse between animals within and between breeds and, therefore, are recommended for pedigree reconstruction based on genetic information;
9. Haplotype blocks: Variants located in haplotype blocks that were not covered by any variant selected in other categories. Using all variants that passed the technical requirements, 145,699 haplotype blocks were generated. All haplotype blocks that were longer than 5 kb or longer than 1 kb but located in the neighborhood of 100 kb of a coding gene were considered. Among the SNPs contributing to the haplotype block, we prioritized those with the highest p-convert score, a MAF > 0.05 in DSN, the highest difference of AAF between DSN and Holstein, and empirical evidence for functioning on the chip from a database of Thermo Fisher Scientific;
10. Filing gaps > 250 kb: Variants filling still existing gaps longer than 250 kb between already selected variants.

Due to a high number of variants in categories 3 and 4, we estimated the linkage disequilibrium (LD) between all these variants with PLINK v.1.9. We kept a maximum of two variants in complete LD within a haplotype block and removed all additional variants in complete LD. As recommended by the manufacturer, oligonucleotides for variants significantly associated with diverse traits of interest (category 2) were added multiple times to ensure their genotype call with higher probability.

### Performance of the DSN200k SNP chip

#### DSN

To test the performance and accuracy of genotyping of DSN cattle using the DSN200k SNP chip, 300 DSN cattle (male = 106, female = 194) were genotyped. 15 of the genotyped DSN cattle had previously been whole genome sequenced (male = 10, female = 5). In addition, we had 16 technical replicates consisting of 2 sequenced DSN males with 4 replicates each, 3 sequenced DSN males with 1 replicate each and 5 sequenced DSN females with 1 replicate each.

Animals were genotyped using the GeneTitan™ Multi-Channel instrument. Genotypes were called using the Axiom® Analysis Suite software v5 following the best practices workflow [53]. The confidence of the called genotype is calculated as *1 - genotype cluster probability* [53] with 0 meaning highest confidence and 1 worst confidence. We removed genotype calls above a confidence of 0.05 (default = 0.15) since higher quality genotypes improve the concordance rate with sequencing data [54]. Furthermore, the call rate of a variant had to exceed the threshold of 0.95 and had to pass QC parameters defined in the best practices [53]. The high-quality variants which passed this filtering are here described as successfully called.

A concordance rate was calculated between genotypes derived from sequencing data and from the DSN200k SNP chip and between genotypes of animals that were genotyped multiple times with the DSN200k SNP chip.

#### Other breeds

We tested the performance of the DSN200k SNP chip for additional cattle populations currently classified as endangered in Germany and Holstein. We genotyped 30 Rotes Höhenvieh (male = 20, female = 10), 30 Angler (male = 30), 30 Rotbunt DN (male = 30), 2 Hinterwälder (male = 2), 12 Original Braunvieh (male = 3, female = 9), 23 Pinzgauer (male = 23), 10 Gelbvieh (male = 4, female = 6), 20 Holstein (male = 10, female = 10). In addition, five sequenced *Bos indicus* Butana cattle (female = 5) were also genotyped with the DSN200k SNP chip. Since all Butana animals were sequenced, the genotypes called from the chip and from sequencing could be compared.

## Supplementary Information

**Additional file 1: Table S1.** Distribution of variants across chromosomes detected by whole genome sequencing and included in the DSN200k chip design. Number of tiled and successfully called variants for DSN are reported.

**Additional file 2: Table S2.** Effects of sequence variants predicted by Ensembl Variant Effect Predictor (VEP) detected in 304 sequenced DSN animals.

**Additional file 3: Figure S1.** Amount of sequence variants selected for the DSN200k SNP chip per chromosome, labeled according to their category of selection.

**Additional file 4: Table S3.** List of all sequence variant IDs in the DSN200k SNP chip.

**Additional file 5: Table S4.** Number of failing variants in all breeds genotyped with the DSN200k SNP chip per category of selection.

**Additional file 6: Table S5.** Number of selected, successfully called (high-quality genotype calls) and in the different populations segregating variants (SNPs and indels) on the DSN200k SNP chip per category of selection.

**Additional file 7: Figure S2.** Number of variants retained in each tranche of truth set variants. The tranche represents the percentage of true variants considered truly true-positives (TPs). In blue are the variants annotated as TPs and in orange as false-positives (FP). Figure generated by VariantRecalibrator tool on GATK v.4.1.3.0.

**Additional file 8: Figure S3.** Histogram of minor allele frequencies (MAFs). The MAFs were binned by 0.05 whereas the lowest MAF was 0.01. Only breeds with at least 5 genotyped animals are shown.

## Data Availability

The datasets generated and analyzed during the current study are publicly available in the European Nucleotide Archive (Whole-sequencing data: PRJEB45822, https://www.ebi.ac.uk/ena/browser/view/PRJEB45822; DSN200k SNP chip data: PRJEB46861, https://www.ebi.ac.uk/ena/browser/view/PRJEB46861).

## Abbreviations

AAF: Alternative Allele Frequency
DSN: Deutsches Schwarzbuntes Niederungsrind
GWAS: Genome Wide Association Studies
ICAR: International Committee for Animal Recording
Illumina BovineSNP50: Illumina BovineSNP50 BeadChip
Indel: Insertion/deletion
ISAG: International Society for Animal Genetics
LD: Linkage Disequilibrium
MAF: Minor Allele Frequency
SNP: Single Nucleotide Polymorphism
VEP: Variant Effect Predictor
WGS: Whole Genome Sequencing
