# Supplementary figures and images for: Design and performance of a bovine 200 k SNP chip developed for endangered German Black Pied cattle (DSN)
Source: BMC Genomics. 2021 Dec 18;22:905. doi: 10.1186/s12864-021-08237-2 (PMC8684242; doi:10.1186/s12864-021-08237-2)

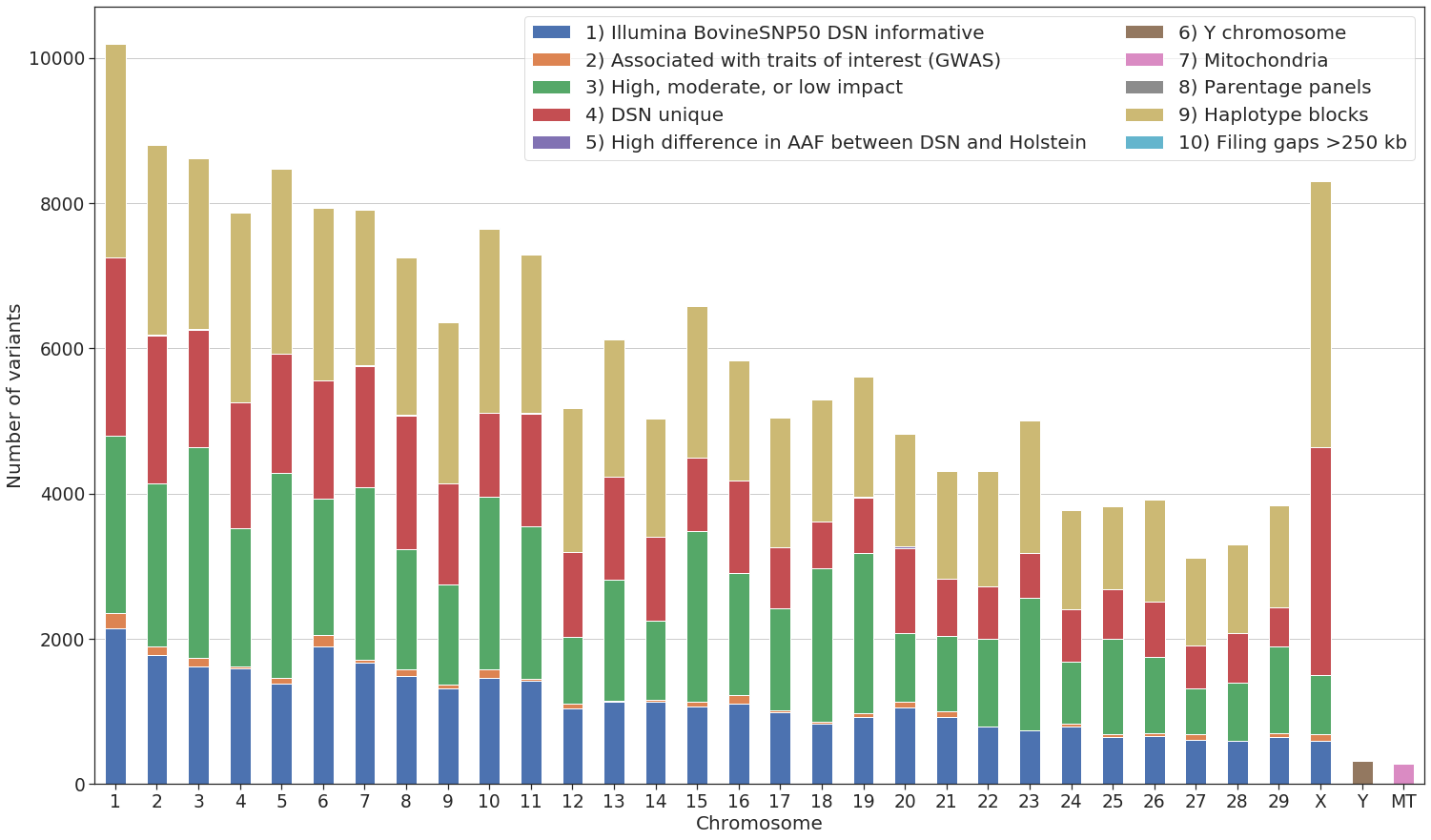

Supplement: Supplementary file 3 — Additional file 3: Figure S1. Amount of sequence variants selected for the DSN200k SNP chip per chromosome, labeled according to their category of selection. [file 12864_2021_8237_MOESM3_ESM.png]

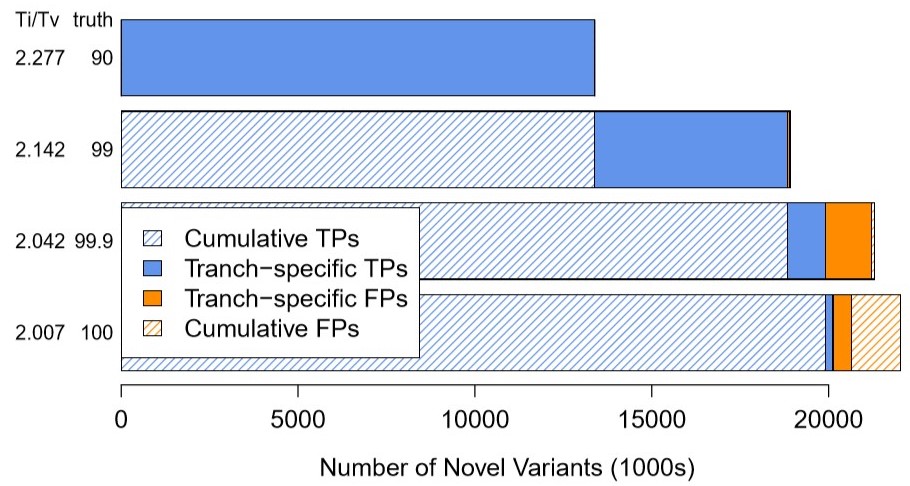

Supplement: Supplementary file 7 — Additional file 7: Figure S2. Number of variants retained in each tranche of truth set variants. The tranche represents the percentage of true variants considered truly true-positives (TPs). In blue are the variants annotated as TPs and in orange as false-positives (FP). Figure generated by VariantRecalibrator tool on GATK v.4.1.3.0. [file 12864_2021_8237_MOESM7_ESM.jpg]

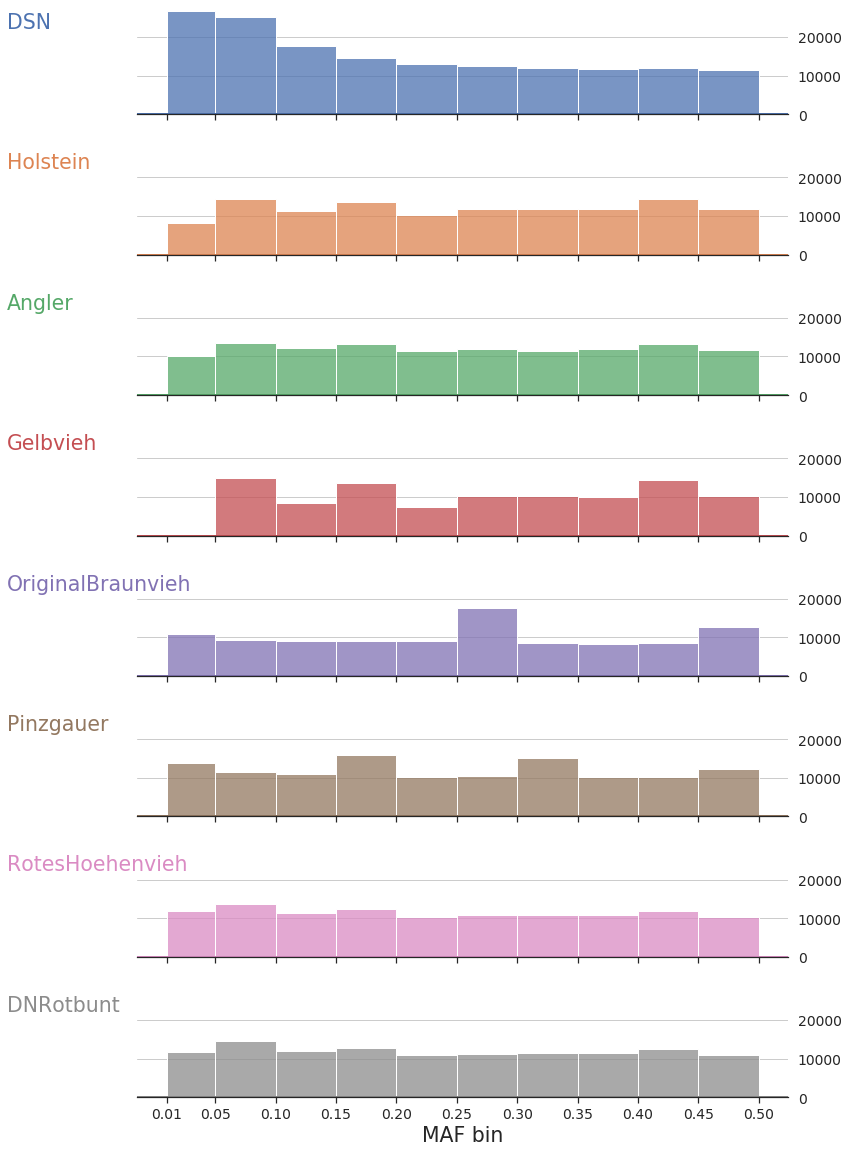

Supplement: Supplementary file 8 — Additional file 8: Figure S3. Histogram of minor allele frequencies (MAFs). The MAFs were binned by 0.05 whereas the lowest MAF was 0.01. Only breeds with at least 5 genotyped animals are shown. [file 12864_2021_8237_MOESM8_ESM.png]
